# Fast Genes and Slow Clades: Comparative Rates of Molecular Evolution in Mammals

**Published:** 2007-05-31

**Authors:** Olaf R. P. Bininda-Emonds

**Affiliations:** Lehrstuhl für Tierzucht, Technical University of Munich, Hochfeldweg 1, 85354 Freising–Weihenstephan, Germany

**Keywords:** Mammalia, molecular-clock hypothesis, molecular evolution, rate shifts, substitution rate

## Abstract

Although interest in the rate of molecular evolution and the molecular clock remains high, our knowledge for most groups in these areas is derived largely from a patchwork of studies limited in both their taxon coverage and the number of genes examined. Using a comprehensive molecular data set of 44 genes (18 nDNA, 11 tRNA and 15 additional mtDNA genes) together with a virtually complete and dated phylogeny of extant mammals, I 1) describe differences in the rate of molecular evolution (i.e. substitution rate) within this group in an explicit phylogenetic and quantitative framework and 2) present the first attempt to localize the phylogenetic positions of any rate shifts. Significant rate differences were few and confirmed several long-held trends, including a progressive rate slowdown within hominids and a reduced substitution rate within Cetacea. However, many new patterns were also uncovered, including the mammalian orders being characterized generally by basal rate slowdowns. A link between substitution rate and the size of a clade (which derives from its net speciation rate) is also suggested, with the species-poor major clades (“orders”) showing more decreased rates that often extend throughout the entire clade. Significant rate increases were rare, with the rates within (murid) rodents being fast, but not significantly so with respect to other mammals as a whole. Despite clear lineage-specific differences, rates generally change gradually along these lineages, supporting the potential existence of a local molecular clock in mammals. Together, these results will lay the foundation for a broad-scale analysis to establish the correlates and causes of the rate of molecular evolution in mammals.

## Introduction

The idea that molecular sequences evolve at a more-or-less constant rate over time (the molecular-clock hypothesis) has underscored research in molecular biology since being proposed for protein sequences by Zuckerkandl and Pauling over 40 years ago (Zuckerkandl and Pauling, 1962, 1965). However, it was clear almost from the outset that no single, global clock exists (see Kumar, 2005). One source of variation in the clock stems from inherent differences in rate among the sequences (genes or proteins) themselves as a result of selection on gene function (Hedges and Kumar, 2003) and mutation rate differences across the genome (Ellegren et al. 2003). A second derives from the later realization that the rate within any single sequence can also vary over time or across lineages (Britten, 1986; Drake et al. 1998). This paper focuses on this second, lineage-dependent source of variation.

Differences in the rate of evolution across the major groups of life are dramatic. For instance, HIV has a substitution rate that is about five orders of magnitude faster than that in mammals (Bromham and Penny, 2003) as a result of the notoriously error-prone DNA replication and proofreading machinery in viruses. Moreover, the extremely high substitution rates in viruses (and other pathogens) might also be maintained by selection, given that they provide a mechanism by which to escape the immune response of the hosts.

Even within a more restricted group such as mammals with its similar molecular machinery, rates differences are still apparent. Two long-standing rules of thumb within mammals are that rodents, and murid rodents in particular, demonstrate an elevated substitution rate (“fast rats”), whereas apes and especially humans have a decreased rate (the “hominid slowdown”) (see Bromham et al. 1996; Kumar, 2005) compared to other mammal species. Other general trends that have been noted for mammals are that whales have generally slow rates (Martin and Palumbi, 1993); that marsupials have slower rates relative to placentals (Martin and Palumbi, 1993); and that the rate in rodents is faster than that in artiodactyls, which in turn is faster than that in primates (see Bromham et al. 1996). These differences have been ascribed variously to slight differences in the efficiency of DNA proofreading and repair enzymes (Hart and Setlow, 1974; Britten, 1986) and any or all of differences in body size, (genome) generation time, mass-specific metabolic rate, or environmental temperature (see Wu and Li, 1985; Li et al. 1987; Martin and Palumbi, 1993; Bromham et al. 1996; Gillooly et al. 2005).

Although many of the general empirical observations in the preceding paragraph are undoubtedly true, apparent discrepancies also occur, such that even widely-accepted findings might not be true universally. For example, Irwin and Arnason (1991) found exactly the opposite trend for “fast rats” and the “hominid slowdown” in *MT-CYB* (better known as cytochrome *b*), with myomorph rodents (as represented by the House Mouse, *Mus musculus*) having the slowest rate and humans and the African Elephant (*Loxodonta africana*) having the highest rate among the 10 sequences (representing eight orders) that they examined. Similarly, Eastal (1991) detected a significant decrease in substitution rate in humans relative to Old World monkeys for only one of the 18 genes he examined (Ψη−globin).

It remains that investigations characterizing comparative rates of molecular evolution in any single group are often based on a highly limited species sample and/or analyses that employed an, at best, limited phylogenetic framework (e.g. using an unresolved star phylogeny). As such, few broad-scale investigations (e.g. Kumar and Subramanian, 2002) have been performed and the observations are usually limited to describing differences in rate between groups, rather than identifying if any rate differences derive from a significant, local rate shift. This study seeks to address this gap in mammals using a comprehensive molecular data set (44 genes comprising 35 427 bp and variously distributed among 2111 species) in concert with a virtually complete dated species-level phylogeny of mammals (Bininda-Emonds et al. 2007) to identify groups with significantly elevated or depressed rates of molecular evolution. Additionally, I present the first attempt to pinpoint the phylogenetic location of any significant changes in rates within mammals. These data will provide an essential foundation to help test between the competing hypotheses (e.g. the metabolic-rate and generation-time hypotheses) of the causal factors influencing molecular evolution in mammals.

## Materials and Methods

### Supertree and gene trees

DNA sequence data from 44 genes were mapped on to suitably pruned versions of the dated mammalian supertree of Bininda-Emonds et al. (2007), which with 4510 species is by far the most complete (99.0% of the species listed in Wilson and Reeder (1993)) and resolved species-level phylogeny for this group. For each gene tree, branch lengths representing the average number of substitutions per site were obtained under a maximum likelihood framework in PAUP* 4.0b10 (Swofford, 2002). In all cases, the most appropriate model of evolution for each gene was determined using the AIC criterion in ModelTEST v3.6 (Posada and Crandall, 1998), albeit with a pruned version of the supertree being used instead of the default NJ tree. Likelihood ratio tests indicated that none of the genes evolved according to a strict molecular clock. Further details regarding both the generation of the gene data sets and trees can be found in Bininda-Emonds et al. (2007).

The 44 genes (18 nDNA, 11 tRNA, and 15 other mtDNA; see Table 1) represent a subset of the 68 genes used to estimate divergence times on the supertree, where each gene included representatives from 10 or more of the orders listed in Wilson and Reeder (1993) (with Artiodactyla and Cetacea combined into Cetartiodactyla, and Insectivora split into Afrosoricida and Eulipotyphla) to ensure broad taxon coverage. There is some inherent circularity in this procedure. However, in both cases, the sequence data were fitted to the same topology under the most appropriate model of evolution, which represents the best estimate of how the data must have evolved. Furthermore, the actual divergence dates were derived from the sequence data of up to 68 genes in combination with 30 fossil calibration points (the latter also representing minimal age constraints), thereby minimizing the circularity for any single gene. A clear benefit to this circularity is that the correspondence between the nodes on the gene trees and the supertree means that all rates of evolution are made in reference to only to those nodes with robust divergence date estimates (i.e. from fossil and/or molecular estimates) and not interpolations from such dates based on relative clade sizes (although the latter could influence the former slightly during the correction for any negative branch lengths). Even so, biases might still occur if there has been a concerted acceleration or deceleration of rates across a whole clade for which the root was dated solely using molecular data. In such cases, the rate change could either not be identified or have its location misidentified.

All 44 genes were protein-coding except for the 11 tRNAs and the mitochondrial genes *MT-RNR1* and *MT-RNR2*. However, sequences for the nuclear-coding genes *APP*, *BMI1*, *CREM*, and *PLCB4* were derived largely or exclusively from untranslated regions flanking the actual coding sequence. I divide the genes into three more-or-less recognizable genomic partitions (nDNA, tRNA, and other mtDNA), largely for convenience. However, reasons exist to suspect rate differences between these partitions. For instance, mtDNA is known to have a higher mutation rate than nDNA, on average, because the mitochondrion is both the source of oxidative phosphorylation in animals (with an increased mutagen production as by-products of metabolic processes) and uses a DNA polymerase-γ with its higher error rate for DNA replication (Bromham and Penny, 2003). Within the mitochondrial genome, tRNAs are distinctly shorter (<100 bps) than the remaining, largely protein-coding genes and are generally held to be relatively conservative evolutionarily.

Both the dated supertree and the sequence data for the 44 genes are freely available on request and can also be found at http://www.uni-jena.de/~b6biol2/PublicationsMain.html/.

### Determining rates of evolution and identifying rate shifts

Because all the nodes in the mammal supertree are dated, it enabled absolute rates of evolution to be calculated for the branches within it rather than the more common and more limited description of relative rates between sister clades in relation to a third group (e.g. as in the relative-rate test of Wu and Li, 1985). For each branch in a given gene tree, the corresponding branch in the mammalian super-tree was determined, with the rate of evolution (number of substitutions per site per year) simply being the length of the branch in the gene tree divided by the duration of the equivalent branch in the supertree. The rate of evolution was associated with the descendant (either a node or a species) of the branch in question. Additionally, cladespecific rates were determined from the branch-specific rates by calculating a series of nested averages, where the rate for a clade was taken to be the arithmetic mean of the rates for all lineages descended from the node subtending that clade. If the descendent lineage was a terminal branch, only the branch-specific rate was used. If the descendant lineage was itself a clade, the rate for the lineage was taken to be the arithmetic mean of the cladespecific rate for the descendent clade and the branch-specific rate between the focal and descendant clades.

In attempting to identify fast- or slow-evolving branches or clades, the assumption is that any global increase or decrease in the evolutionary rate will be mirrored consistently across all genes for a given branch or clade. When comparisons were made across all genes, two procedures were used to compensate for rates of molecular evolution being gene-specific and therefore often differing greatly. First, all individual absolute rates were log-transformed (base *e*) to correct for any large differences in gene-specific rates that would bias parametric (paired) statistical tests. In so doing, a correction needs to be made for rates of magnitude zero (for which the logarithm is undefined), which involved adding the exponent of a given rate to its raw value. Thus, for example, the corrected value for a rate of 1.68 × 10^−9^ was ln(1.68 × 10^−9^ + 10^−9^). When the rate to be tested itself was zero, the exponent from the rate it was being compared to was added instead. Second, all comparisons were paired, such that the tested (ln-transformed) rate for a given gene was only compared to some reference (ln-transformed) rate for the same gene. The use of pairwise comparisons also accounts for any gene-specific differences, such as differences in base composition or GC content, which would otherwise necessitate the removal of the heterogeneous genes from the analysis (e.g. Kumar and Subramanian, 2002), a procedure that has been argued to be unjustified (Ellegren et al. 2003). Note that these corrections, and the second in particular, were performed without regard to the genomic partition to which a gene belong (i.e. nDNA, tRNA, or other mtDNA).

Together, these two corrections ensure that the scale of the difference between the rates being compared and not their magnitude is of primary importance. For both branch- and cladespecific rate investigations, both paired parametric (Student’s *t*-test) and nonparametric (Fisher’s sign test) two-tailed statistical analyses were used, with a nominal α = 0.05. Corrections for multiple comparisons employed a sequential Bonferroni technique (Rice, 1989).

Essentially, the branch-specific analyses attempt to identify localized rate changes, perhaps associated with a major adaptive event (e.g. an adaptive radiation or ecological transition) or a severe environmental disturbance likewise engendering a rapid adaptive response. By contrast, the cladespecific analyses attempt to identify entire clades with an altered evolutionary rate, even if no single branch within or leading to that clade displays a significant rate shift. To identify fast- and slow-evolving branches or clades, the respective tested rates were compared to one of two reference rates for a given gene: 1) that representing the average rate across mammals (= the cladespecific rate of the root node of the gene tree) or 2) that representing an ancestral node on the supertree that was no more than three intervening branches removed from the target node. The former set of “rate-outlier” analyses attempt to identify branches or clades with substitution rates that differ significantly from the global mammalian average, whereas the latter “rate-shift analyses” attempt to pinpoint where any significant, local changes in rate have occurred. For instance, the fact that a number of closely neighbouring branches or clades are all identified as (global) rate outliers could result from a single rate shift in the oldest branch or clade, with the new rate being inherited by the descendent lineages. Moreover, the rate-shift analyses can also identify branches or clades with rates that differ significantly from the local value, even though they might not differ significantly from the global mammalian average,

In both the rate-outlier and rate-shift analyses, fast- or slow evolving branches or clades were defined as those where either the average (arithmetic mean) paired difference over all genes compared to the reference node differed significantly from zero (paired *t*-test) or the proportion of positive comparisons differed significantly from 0.5 (paired sign test). For the rate-shift analyses, the ancestral reference node was taken to be the one yielding the greatest number of paired comparisons or was the closest to the target node in the case of a tie. For presentation purposes, the proportion of values underlying the sign test is presented as (*n*_+_ − *n*_−_)/(*n*_+_ + n_−_), such that proportions of 1 and −1 indicate all positive and all negative comparisons, respectively.

The methods and tests described in this section (apart from any corrections for multiple comparisons) have all been implemented in the Perl script moleRat v1.0, which is freely available at http://www.uni-jena.de/~b6biol2/ProgramsMain.html/. In this study, the default values for the program were used, including the option to ignore all branches in the gene trees with a length of less than 0.0001 substitutions per site per unit time.

## Results

### Gene-specific rates of evolution

**Figure 1:**
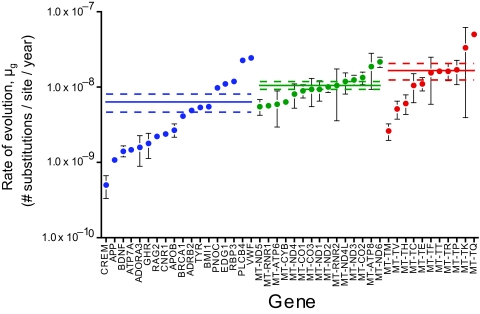
Absolute rates of molecular evolution for 44 different genes. Gene are localized to their genomic partition (nDNA, blue; other mtDNA, green; tRNA, red) and are presented in increasing order of rate. Error bars represent SEs and, when not visible, are subsumed within the plot symbol. Solid and dashed lines represent the average rate ± SE for the respective partition.

The average absolute rates of evolution for the 44 genes (Figure 1) are generally on an order of magnitude of either 10^−8^ or 10^−9^ substitutions per site per year and range between 4.92 × 10^−10^ (*CREM*) and 4.95 × 10^−8^ (*MT-TQ*), a difference of nearly two full orders of magnitude. These values agree broadly with those published previously, although they are significantly higher than the mammalian average of 2.2 × 10^−9^ calculated across 5669 genes by Kumar and Subramanian (2002) (one-sample Student’s *t* = 5.70, *df* = 43, *p* < 0.0001). Rates for genes comprising primarily untranslated regions of coding genes were distributed throughout the nuclear genes, including the two slowest rates (*APP* and *CREM*) and one of the fastest (*PLCB4*).

An ANOVA revealed a significant difference in rate between the genomic partitions (F_2,41_ = 4.883, *p* = 0.0125), with Fisher’s PSLD test showing that nDNA is evolving significantly slower than tRNA (by 2.6× *p* = 0.0033). In fact, despite being widely perceived as being conserved evolutionarily, tRNA genes exhibited the fastest rates of all partitions on average, although they were not significantly different from those of the remaining mtDNA genes (1.6 × faster; *p* = 0.0783), which, in turn, were not significantly different from those of the nDNA genes (1.6 × faster; *p* = 0.1783). However, these observations do not exclude the possibility that the unexpectedly higher rates in tRNA genes derive primarily from substitutions concentrated in hypervariable regions or are due to stochastic variation arising from the extremely short sequence lengths (<100 bp). In the latter case, however, there is no reason why any stochastic variation would cause all tRNA genes to show such a relative uniformity in rate, or at least not one any appreciably greater than for nDNA and other mtDNA genes.

### Branch-specific rates of evolution

**Figure 2:**
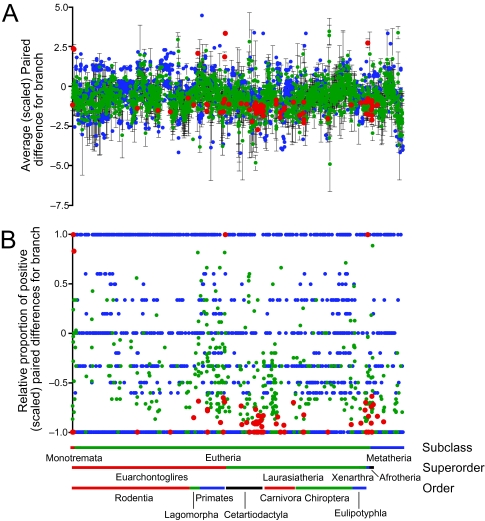
Branch-specific rates of evolution in mammals (outlier rates). Rates were evaluated with either (a) a *t* -test or (b) a sign test (red = significantly fast / slow at a nominal alpha of 0.05; green = not significant; blue = insufficient sample size for testing). In (a), values represent average paired difference (± SE) between the target branch and the gene-specific rate for all relevant genes.

Rate estimates were available for 1246 of the internal and 2086 of the terminal (species) branches of the supertree, or about half (50.3%) of all 6618 branches. Across the entire tree, slowdowns in outlier rates are more common (Figure 2): average paired difference ± SE = −0.571 ± 0.018 (*n*_+_ = 815; *n*_−_ = 2517; *n*_0_ = 0) and average proportion ± SE = −0.378 ± 0.013 (*n*_+_ = 773; *n*_−_ = 2270; *n*_0_ = 289). Only six branches exhibit a rate that is significantly faster than the mammalian average (summarized in Table 2). All six subtend clades, most of which characterized major lineages comprising two or more orders (the two exceptions being the branches leading to Bovidae and Hystricomorpha + Myomorpha). Many more branches were indicated to have significantly slow rates of evolution, including the branch leading to Monotremata and most branches within this clade, the branches leading to each of the great ape species except the Orangutan *Pongo pygmaeus*, two major clades within mysticete whales as well as numerous individual cetacean species, the branch leading to Perissodactlya and numerous branches within this order, and several branches leading to or within Afrotheria and Xenarthra (Table 2). In fact, the majority of the slow branches identified (55 of 68 for the paired *t*-test; 52 of 70 for the paired sign test) were terminal ones leading to individual species, indicating that the potential confounding of the mutation and substitution rates (sensu Ho and Larson 2006) was not a problem here.

**Figure 3:**
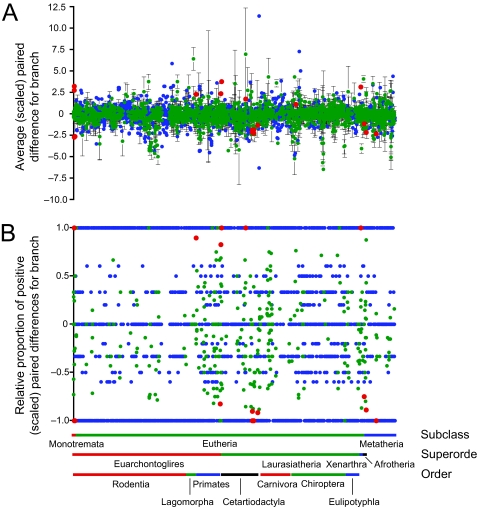
Branch-specific rates of evolution in mammals (rate shifts). Rates were evaluated with either (a) a *t* -test or (b) a sign test (red = significantly fast / slow at a nominal alpha of 0.05; green = not significant; blue = insufficient sample size for testing). In (a), values represent average paired difference (± SE) between the target branch and an ancestral branch.

Few local shifts in branch-specific rates were detected among the 3243 branches with a suitable reference branch (summarized in Tables 3 and 4). Again, rate slowdowns were more common across the tree, although not to the same degree as for the outlier rates (Figure 3): average paired difference ± SE = −0.150 ± 0.019 (*n*_+_ = 1419; *n*_−_ = 1824; *n*_0_ = 0) and average proportion ± SE = −0.070 ± 0.014 (*n*_+_ = 1210; *n*_−_ = 1530; *n*_0_ = 503). Rate-shift analyses confirmed that all fast outlier branches also represent significant local rate shifts. Three additional local speedups were also indicated in the branches leading to Cetartiodactyla + Perissodactyla, Delphinidae + Phocoenidae within Cetacea, and Microchiroptera. Significant local slowdowns were concentrated in Cetacea, but also occurred along the branches leading to Boreoeutheria, Tubulidentata, Xenarthra, and, most interestingly, the rodent families Dipododae + Muridae. These results did not change appreciably when the rate-shift analyses were constrained such that the ancestral, reference branch was the immediate ancestor of the target branch (compare Tables 3 and 4).

### Clade-specific rates of evolution

**Figure 4:**
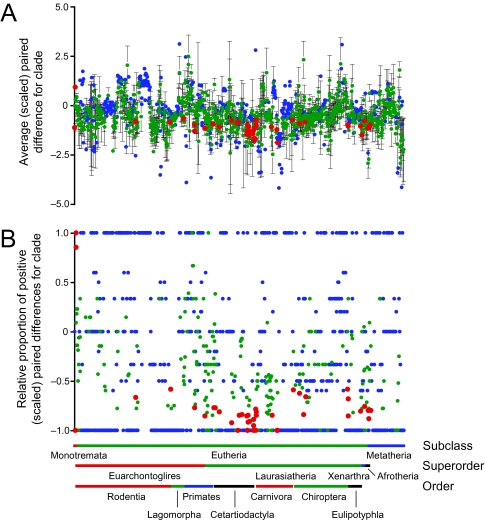
Clade-specific rates of evolution in mammals (outlier rates). Rates were evaluated with either (a) a *t* -test or (b) a sign test (red = significantly fast / slow at a nominal alpha of 0.05; green = not significant; blue = insufficient sample size for testing). In (a), values represent average paired difference (± SE) between the target clade and the gene-specific rate for all relevant genes.

Clade-specific rate estimates were present for 1282 of the 2108 nodes (60.8%) on the mammal super-tree. As for the branch-specific rates, the overall trend is for a predominance of rate slowdowns in the outlier rates (Figure 4): average paired difference ± SE = −0.421 ± 0.025 (*n*_+_ = 340; *n*_−_ = 941; *n*_0_ = 1) and the average proportion ± SE = −0.323 ± 0.021 (*n*_+_ = 327; *n*_−_ = 834; *n*_0_ = 120). Clades identified as significant rate outliers (Table 5) generally reflect the results of the branch-specific analyses. Important slow clades include Monotremata and Tachyglossidae, the clade Cetartiodactyla + Perissodactyla and numerous clades within each order, squirrel-like rodents (Sciuromorpha), the hominoid clades *Homo* + *Pan* and *Pan*, and several major clades in Carnivora and in the superorders Afrotheria and Xenarthra. The traditional orders seem to be disproportionately characterized as being significantly slow, with examples including Afrosoricida, Carnivora, Chiroptera, Eulipotyphla (albeit excluding Soleonodontidae), Lagomorpha, Marsupialia, Monotremata, Perissodactyla, and Xenarthra. The only fast clades compared to mammals as a whole were Theria (= Eutheria + Marsupialia) and Eutheria.

**Figure 5:**
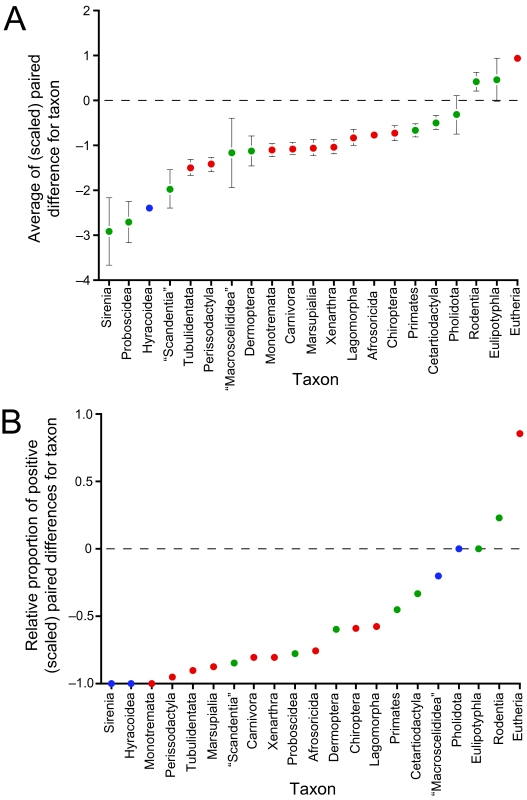
Clade-specific rates of evolution for selected clades of mammals (outlier rates). Rates were evaluated with either (a) a *t*-test or (b) a sign test (red = significantly fast / slow at a nominal alpha of 0.05; green = not significant; blue = insufficient sample size for testing). The dashed line indicates the average value across mammals. In (a), values represent average paired difference (± SE) between the target clade and the gene-specific rate for all relevant genes. The rates for the clades labeled “Macroscelididae” and “Scandentia” actually represent those for Macroscelididae without Rhynchocyon and Tupaiinae, respectively.

The latter observations are underscored more strongly by the restricted view in Figure 5 that reveals that nearly all the major mammalian lineages — generally, the orders, with the addition of the branch-specific outlier rate for the monotypic Tubulidentata (= *Orycteropus afer*) — show slower rates, and usually significantly slower rates, than do mammals as a whole. The only “fast” clades are Eutheria, Rodentia, and, as indicated by the paired *t*-test only, Eulipotyphla. Only the increased rate for Eutheria was significantly increased (average paired difference = 0.924) and, interestingly, mirrored the magnitude of the significantly decreased rates characterizing its sister clade, Marsupialia (−1.060), nearly exactly in magnitude.

**Figure 6:**
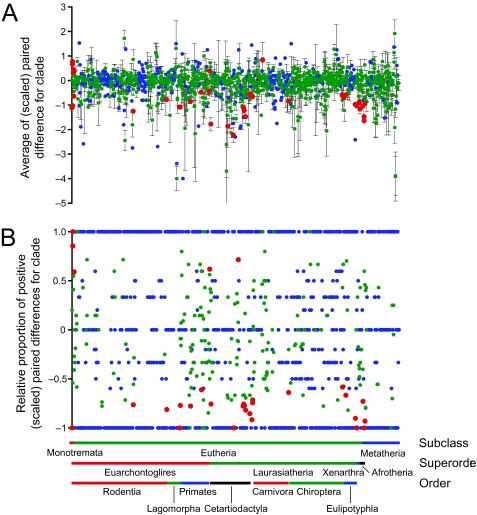
Clade-specific rates of evolution in mammals (rates shifts). Rates were evaluated with either (a) a *t* -test or (b) a sign test (red = significantly fast / slow at a nominal alpha of 0.05; green = not significant; blue = insufficient sample size for testing). In (a), values represent average paired difference (± SE) between the target clade and an ancestral clade.

The rate-shift analyses confirm that most fast and slow rate-outlier clades also represent instances of significant local rate changes (Figure 6; Tables 6 and 7). Significant local increases were also found for Boreoeutheria, Rodentia, the clade of sea lions in Carnivora, a major clade within Cetacea, and Cetartiodactyla as a whole. Important local slowdowns include the clades of Boreoeutheria + Xenarthra (compared to the fast Eutheria) and Myomorpha + Hystricomorpha (compared to the fast Rodentia); both instances apparently derive from the fast branch-specific rates associated with each clade. Finally, the hominoid clade of *Gorilla* + *Homo* + *Pan* as a whole, but no clades within it, was inferred to have undergone a local slowdown. The more restrictive rate-shift analyses (Table 7) largely confirmed this general pattern, although they identified only a subset of the clades inferred to have undergone a significant shift in the cladespecific rate of evolution. Altogether, many of the groups displaying rate shifts represent classic mammalian orders or major groupings thereof, suggesting a slowdown in the rate of molecular evolution following their establishment and initial diversification. Otherwise, rate-shifts in cladespecific rates across the tree showed the same tendency towards local slowdowns as seen in the other analyses: average paired difference ± SE = −0.155 ± 0.017 (*n*_+_ = 558; *n*_−_ = 703; *n*_0_ = 2) and the average proportion ± SE = −0.055 ± 0.022 (*n*_+_ = 491; *n*_−_ = 599; *n*_0_ = 171).

## Discussion

Overall, the results indicate that the rate of molecular evolution across many genes considered simultaneously is relatively homogeneous among mammals, with comparatively few significant outlier rates or rate shifts being detected for both branch- and cladespecific rates. A similar conclusion was reached by Kumar and Subramanian (2002), albeit with fewer taxa (326) but many more genes (5669). Together, these findings could be taken as evidence supporting a local (but not global) molecular clock (at least for mammals), an idea that at least implicitly underlies many of the relaxed molecular clock methods currently being used to derive divergence times from molecular data (for recent reviews, see Renner, 2005; Welch and Bromham, 2005). However, these findings also do not exclude the possibility that rates are changing substantially and frequently, but only among selected genes in selected lineages (e.g. Smith and Eyre-Walker, 2003). Such changes, naturally, point to a gene-specific, selection-driven alteration in rate, rather than to a global change in the rate of molecular evolution between lineages associated with differences in any or all of body size, (genome) generation time, mass-specific metabolic rate, or environmental temperature.

The few significant differences in rate observed largely corroborate the previous general conclusions of other workers. For instance, as noted by Martin and Palumbi (1993), whales were confirmed as generally being characterized by a slow substitution rate, whether for entire clades or numerous individual species. Several shifts to even slower rates of evolution within Cetacea were also observed. Similarly, there is good support for progressive local rate decreases within hominids across a large number of genes (contra Eastal, 1991), thereby supporting the existence of the “hominid slowdown” (see Bromham et al. 1996; Kumar and Hedges, 1998). Marsupials were also shown to have a slower rate compared to placental mammals (Martin and Palumbi, 1993) and, in fact, both taxa differed significantly from the overall mammalian average in their cladespecific rates (slower and faster, respectively).

The broad taxon sampling in this study also allowed the identification of several apparently novel trends, including general rate slowdowns in monotremes, perissodactyls, and various afrotherian taxa. Moreover, there appears to be a tendency for rate slowdowns to be concentrated basally among the orders or major mammalian lineages; apart from Cetacea and hominids, few rate slowdowns were associated with species or more terminal clades in the tree. The more depauperate major lineages (e.g. Afrotheria, Perissodactyla or Xenarthra) also seem to be characterized by more systemic slowdowns occurring throughout the clade, suggesting a possible link between the rate of molecular evolution and the net rate of speciation. This supposition is supported by the observation of weak, but significant relationships between the ln-transformed sizes of the orders in Figure 5 (which yield non-nested and therefore reasonably phylogenetically independent data points) and their clade-specific rates of evolution as given by either the parametric paired difference (*p* = 0.0012, *df* = 19, *R*^2^ = 0.449) or the non-parametric proportion of positive values (*p* = 0.0285; *df* = 19, *R*^2^ = 0.240). For the former set of analyses at least, this relationship still holds even when the Rodentia are excluded as a potential outlier. Although the relationship here deals with molecular rates and not total amount of molecular change, it still agrees with the predictions of Pagel et al. (2006) and so might support their arguments for an increased role for punctuational effects in speciation. It cannot be excluded, however, that the significant association derives at least in part from the known node-density artifact where the degree of molecular change is apparently increased in those parts of the tree with denser taxon sampling (Fitch and Beintema, 1990; Webster et al. 2003), although the use of maximum likelihood to derive the gene trees under the inferred optimal model of evolution should mitigate any negative effects (Venditti et al. 2006).

An unexpected result given the widespread acceptance of the “fast-rats” hypothesis was that few significantly increased substitution rates were found at any level within rodents. At best, only two significant rate increases were observed, neither of which were associated directly with murids: a fast outlier rate for the branch leading to Hystricomorpha + Myomorpha (the latter of which does contain Muridae, however) and a cladespecific rate shift for rodents as a whole. However, rodents did possess among the fastest rates of all the orders examined and are generally characterized by increased outlier rates (e.g. see Figure 5), and for both branches and clades, just not significantly increased ones. Previous evidence for an elevated rate of evolution for (murid) rodents also derives largely from specific, pairwise comparisons with other, slower groups (such as primates), thereby accentuating lineage-specific differences and not the more global and local perspectives examined here. Interestingly, Kumar and Subramanian (2002) also show that rate differences within each of primates and rodents are of similar magnitudes to those between the two taxa, indicating that that apparent rate increase in rodents may have been overstated or is dependent on the species being investigated.

Thus, the general lack of any significant rate shifts within rodents appears to indicate a real lack of any dramatic local changes in the substitution rate within the group. However, it cannot be excluded that the result is a partial artifact of the high substitution rates in rodents causing the divergence time estimates in this clade being too old (see Steppan et al. 2004), thereby causing the inferred substitution rates to be underestimated. Indeed, the divergence time in the supertree for the split between the murid genera *Mus* and *Rattus* of 30.3 million years ago (mya) is over three times that advocated by Steppan et al. (2004) based on paleontological evidence (8.8 – 10.3 mya). This problem would not affect pairwise comparisons between rodents and another group, where divergence time would be factored out because both lineages would be equally old.

Interestingly, the observations of Irwin and Arnason (1991) with respect to the “inverted” relative substitution rates in *MT-CYB* were upheld partly here. In particular, the three hominoid primates *Homo sapiens*, *Pan paniscus*, and *Pan troglodytes* did indeed all possess higher rates of evolution for this gene (8.16 × 10^−9^, 6.07 × 10^−9^, and 9.88 × 10^−9^ substitutions per site per year, respectively) than did the rodents *Mus musculus* and *Rattus novegicus* (4.78 × 10^−9^ and 5.23 × 10^−9^, respectively). The latter pair of rates also fell below the overall rate for *MT-CYB* of 6.37 × 10^−9^ substitutions per site per year. *Loxodonta africana*, however, displayed the slowest rate for this gene among the relevant species at 3.85 × 10^−9^ substitutions per site per year (contra Irwin et al. 1991).

Finally, some evidence of non-independent rate shifts exists. For instance, the shifts to a slower rates for the branches leading to either Myomorpha or Muridae + Dipodidae derive from these branches being compared to the rate for the branch leading to Hystricomorpha + Myomorpha, a significantly fast branch. So, although the rate slowdown is perhaps unexpected here, it would only be in a global sense; these results otherwise seem to reflect local events accurately. More importantly, there does not seem to be much evidence of the truly artifactual “trickle-down effect” (sensu Moore et al. 2004), whereby a large outlier rate for a clade is passed down the tree to its parent clade. Instances of congruent significant outlier rates among linked clades are present in Table 5 (e.g. within Carnivora, Cetacea, or Perissodactyla), but the rate for the parental clade is often more significant than that for the daughter clade, indicating an additive effect of the sister clades. Under a trickle-down scenario, the effect would be expected instead to dissipate progressively going up the tree.

## Conclusions

The comparative paucity of significant rate differences observed in this study cannot be taken to mean that lineage-specific differences are largely absent among mammals, simply that few differences exist with respect to either the overall mammalian average (outlier-rate analyses) or from a local reference point (rate-shifts analyses). Systematic, significant differences in rate could still exist between specific lineages, such as between rodents and primates for example (see also Figure 5), and perhaps also restricted to specific genes (e.g. Smith and Eyre-Walker 2003).

This fact is underscored by the large differences in the rate of evolution that are apparent here. Among those values for average paired differences in rate that could be tested significantly (i.e. paired *n* >1), the fastest branch was that leading to the node joining the bat genera *Molossus* and *Promops* (3.47), whereas the slowest was that leading to the Black Mastiff Bat, *Molossus ater* (−4.91), a species within the former clade. The respective values for cladespecific rates are less extreme, but still dramatic, with the fastest and slowest clades being a clade of five *Macaca* species (2.51) and the species pair of *Didelphis aurita* and *Didelphis marsupialis* (−2.93), respectively. Differences in rate within any single gene are even more dramatic, with the difference between the slowest and fastest branch-specific rate for a given gene ranging between 114× (*TYR*) and 1.12 × 10^9^× (*MT-TF*) (results not shown).

Despite concerted effort, the reasons underlying any global lineage-specific differences remain unclear, with explanations invoking or refuting any or all of differences in cellular DNA proofreading and repair mechanisms, body size, mass-specific metabolic rate, and/or (genomic) generation time (for a recent review, see Kumar and Hedges, 1998). The current data set, together with a database containing relevant trait data for a large number of mammal species (http://www.biodiversitydata.group.cam.ac.uk/pantheria/pantheria.html), will allow for a more broadly-based, phylogenetic analysis than has been possible before now, thereby providing key insights into the correlates and causes of global differences in the rate of molecular evolution.

## Figures and Tables

**Table 1:** Genes included for analysis from the data set of Bininda-Emonds et al. (2007) and relevant statistics. “Taxon coverage” refers to the number of orders listed in Wilson and Reeder (1993) (with Artiodactyla and Cetacea combined into Cetartiodactyla, and Insectivora split into Afrosoricida and Eulipotyphla) for which sequences were available. Gene names are standardized according to the Human Genome Nomenclature Committee names (Wain et al. 2002).

**Gene**	**Genome**	**Taxa**	**Length**	**Taxon coverage**	**Model**
MT-ATP6	mtDNA	200	708	12	GTR + I + G
*MT-ATP8*	mtDNA	190	213	13	GTR + I + G
*MT-CO1*	mtDNA	221	1563	14	GTR + I + G
*MT-CO2*	mtDNA	413	711	15	GTR + I + G
*MT-CO3*	mtDNA	281	858	13	GTR + I + G
*MT-CYB*	mtDNA	1290	1200	17	GTR + I + G
*MT-ND1*	mtDNA	364	969	16	GTR + I + G
*MT-ND2*	mtDNA	282	1068	15	TVM + I + G
*MT-ND3*	mtDNA	324	360	12	GTR + I + G
*MT-ND4*	mtDNA	322	1461	14	GTR + I + G
*MT-ND4L*	mtDNA	351	297	13	GTR + I + G
*MT-ND5*	mtDNA	165	1857	15	GTR + I + G
*MT-ND6*	mtDNA	153	558	13	GTR + I + G
*MT-RNR1*	mtDNA	813	1160	20	GTR + I + G
*MT-RNR2*	mtDNA	742	2677	19	GTR + I + G
*ADORA3*	nDNA	77	330	13	TrN + I + G
*ADRB2*	nDNA	80	1263	13	TVM + I + G
*APOB*	nDNA	76	1350	17	GTR + I + G
*APP*	nDNA	70	806	13	GTR + G
*ATP7A*	nDNA	74	690	13	TIM + I + G
*BDNF*	nDNA	96	804	15	K81uf + I + G
*BMI1*	nDNA	64	345	11	GTR + G
*BRCA1*	nDNA	149	3130	16	TVM + I + G
*CNR1*	nDNA	91	1098	11	TVM + I + G
*CREM*	nDNA	72	476	12	TVM + I + G
*EDG1*	nDNA	69	978	13	TVM + I + G
*GHR*	nDNA	146	2016	12	TVM + I + G
*PLCB4*	nDNA	74	410	13	TIM + I + G
*PNOC*	nDNA	74	585	13	TVM + I + G
*RAG2*	nDNA	219	1584	11	TVM + I + G
*RBP3*	nDNA	547	1302	16	GTR + I + G
*TYR*	nDNA	76	426	12	SYM + I + G
*VWF*	nDNA	190	1276	17	TVM + I + G
*MT-TR (tRNA-ARG)*	tRNA	266	75	10	TVM + G
*MT-TC (tRNA-CYS)*	tRNA	138	83	10	K81uf + I + G
*MT-TQ (tRNA-GLN)*	tRNA	117	79	10	HKY + I + G
*MT-TE (tRNA-GLU)*	tRNA	120	75	11	GTR + I + G
*MT-TH (tRNA-HIS)*	tRNA	274	74	10	TVM + I + G
*MT-TK (tRNA-LYS)*	tRNA	127	82	10	TrN + I + G
*MT-TM (tRNA-MET)*	tRNA	127	74	11	GTR + I + G
*MT-TF (tRNA-PHE)*	tRNA	200	85	11	TrN + G
*MT-TP (tRNA-PRO)*	tRNA	317	125	13	TVM + I + G
*MT-TT (tRNA-THR)*	tRNA	222	88	11	TVM + I + G
*MT-TV (tRNA-VAL)*	tRNA	648	94	20	TIM + I + G

**Table 2:** Branches identified as being significant rate outliers compared to mammals as a whole. All *P*-values listed are significant at a nominal alpha of 0.05 corrected for multiple comparisons.

**Node**	**Order**	**Description**	**Paired*n***	**Average paired difference**	**SE**	**Paired *t***	**Paired*t P-*value**	**n_+_**	**n_−_**	**Proportion**	**Paired sign*P*-value**
Node 3	Monotremata	Tachyglossidae	15	−1.16	0.21	−5.56	ns	0	15	−1.00	6.10 × 10^−5^
*Zaglossus bruijni*	Monotremata		15	−1.25	0.29	−4.28	ns	0	15	−1.00	6.10 × 10^−5^
*Ornithorhynchus anatinus*	Monotremata		17	−1.17	0.16	−7.23	1.99 × 10^−6^	0	17	−1.00	1.53 × 10^−5^
Node 6		Boreoeutheria + Xenarthra	22	2.40	0.19	12.80	2.61 × 10^−11^	22	0	1.00	4.77 × 10^−7^
Node 11	Rodentia	Myomorpha + Hystricomorpha	24	2.36	0.29	8.20	2.83 × 10^−8^	22	2	0.83	3.59 × 10^−5^
*Castor canadensis*	Rodentia		20	−1.41	0.21	−6.74	1.93 × 10^−6^	0	20	−1.00	1.91 × 10^−6^
*Erethizon dorsatum*	Rodentia		19	−1.54	0.24	−6.32	5.87 × 10^−6^	2	17	−0.79	ns
*Tamias striatus*	Rodentia		17	−1.63	0.25	−6.47	7.80 × 10^−6^	0	17	−1.00	1.53 × 10^−5^
Node 673	Lagomorpha	Lagomorpha	39	−0.75	0.15	−4.88	1.92 × 10^−5^	8	31	−0.59	ns
*Oryctolagus cuniculus*	Lagomorpha		27	−1.19	0.19	−6.43	8.28 × 10^−7^	2	25	−0.85	5.65 × 10^−6^
Node 749		Primates + Dermoptera + Scandentia	22	2.08	0.32	6.61	1.51 × 10^−6^	20	2	0.82	ns
Node 753	Primates	Simiiformes	38	−0.84	0.18	−4.75	ns	6	32	−0.68	2.43 × 10^−5^
*Gorilla gorilla*	Primates		27	−1.11	0.21	−5.21	1.94 × 10^−5^	4	23	−0.70	ns
*Homo sapiens*	Primates		44	−1.56	0.21	−7.53	2.24 × 10^−9^	5	39	−0.77	1.41 × 10^−7^
*Pan paniscus*	Primates		22	−0.71	0.15	−4.70	1.23 × 10^−4^	0	22	−1.00	ns
*Pan troglodytes*	Primates		28	−1.17	0.18	−6.56	4.86 × 10^−7^	3	25	−0.79	2.74 × 10^−5^
*Lemur catta*	Primates		40	−1.64	0.20	−8.41	2.73 × 10^−10^	2	38	−0.90	1.49 × 10^−9^
Node 921	Dermoptera	Dermoptera	15	−1.62	0.23	−6.99	6.30 × 10^−6^	0	15	−1.00	6.10 × 10^−5^
*Cynocephalus variegatus*	Dermoptera		40	−1.06	0.19	−5.71	1.32 × 10^−6^	7	33	−0.65	4.23 × 10^−5^
Node 930		Cetartiodactyla + Perissodactyla + Carnivora + Pholidota	22	1.88	0.30	6.30	2.99 × 10^−6^	20	2	0.82	ns
Node 932	Cetartiodactyla	Cetartiodactyla	33	−0.82	0.16	−4.99	2.06 × 10^−5^	5	28	−0.70	6.62 × 10^−5^
Node 938	Cetartiodactyla	Bovidae	18	3.32	0.18	18.60	4.24 × 10^−12^	18	0	1.00	7.63 × 10^−6^
*Ovis aries*	Cetartiodactyla		24	−1.11	0.16	−6.75	7.00 × 10^−7^	3	21	−0.75	ns
*Bos taurus*	Cetartiodactyla		30	−1.09	0.19	−5.83	2.57 × 10^−6^	4	26	−0.73	5.95 × 10^−5^
*Bubalus bubalis*	Cetartiodactyla		26	−1.65	0.61	−2.70		1	25	−0.92	8.05 × 10^−7^
*Muntiacus crinifrons*	Cetartiodactyla		21	−1.14	0.20	−5.74	1.28 × 10^−5^	1	20	−0.91	2.10 × 10^−5^
*Phocoena phocoena*	Cetartiodactyla		24	−1.50	0.25	−6.10	3.20 × 10^−6^	0	24	−1.00	1.19 × 10^−7^
*Tursiops truncatus*	Cetartiodactyla		14	−2.22	0.32	−6.95	1.01 × 10^−5^	1	13	−0.86	ns
*Lagenorhynchus albirostris*	Cetartiodactyla		23	−1.12	0.23	−4.82	ns	1	22	−0.91	5.72 ×10^−6^
*Monodon monoceros*	Cetartiodactyla		25	−1.27	0.17	−7.64	7.06 × 10^−8^	0	25	−1.00	5.96 × 10^−8^
*Berardius bairdii*	Cetartiodactyla		24	−1.32	0.20	−6.56	1.07 × 10^−6^	1	23	−0.92	2.98 × 10^−6^
*Hyperoodon ampullatus*	Cetartiodactyla		22	−1.42	0.20	−7.11	5.21 × 10^−7^	1	21	−0.91	1.10 × 10^−5^
*Platanista minor*	Cetartiodactyla		26	−1.04	0.17	−6.19	1.77 × 10^−6^	3	23	−0.77	8.80 × 10^−5^
*Kogia breviceps*	Cetartiodactyla		20	−1.18	0.21	−5.54	2.40 × 10^−5^	1	19	−0.90	4.01 × 10^−5^
*Physeter catodon*	Cetartiodactyla		26	−1.48	0.22	−6.82	3.76 × 10^−7^	0	26	−1.00	2.98 × 10^−8^
Node 1083	Cetartiodactyla	Balaenopteridae ~ Balaenidae +	19	−1.25	0.26	−4.74		1	18	−0.90	7.63 × 10^−5^
*Balaena mysticetus*	Cetartiodactyla		21	−1.70	0.27	−6.25	4.17 × 10^−6^	1	20	−0.91	2.10 × 10^−5^
*Caperea marginata*	Cetartiodactyla		24	−1.28	0.16	−8.08	3.64 × 10^−8^	1	23	−0.92	2.98 × 10^−6^
Node 1087	Cetartiodactyla	*Baleonoptera* + *Megaptera* + *Eschrichtius*	17	−0.91	0.21	−4.40	ns	0	17	−1.00	1.53 × 10^−5^
*Balaenoptera acutorostrata*	Cetartiodactyla		21	−1.48	0.18	−8.25	7.18 × 10^−8^	1	20	−0.91	2.10 × 10^−5^
*Balaenoptera musculus*	Cetartiodactyla		19	−1.64	0.14	−11.60	8.30 × 10^−10^	0	19	−1.00	3.81 × 10^−6^
*Balaenoptera physalus*	Cetartiodactyla		20	−1.45	0.19	−7.80	2.43 × 10^−7^	1	19	−0.90	4.01 × 10^−5^
*Megaptera novaeangliae*	Cetartiodactyla		17	−2.75	0.26	−10.60	1.18 × 10^−8^	0	17	−1.00	1.53 × 10^−5^
*Eschrichtius robustus*	Cetartiodactyla		24	−1.58	0.22	−7.23	2.32 × 10^−7^	2	22	−0.83	3.59 × 10^−5^
*Hippopotamus amphibius*	Cetartiodactyla		39	−1.45	0.17	−8.67	1.55 × 10^−10^	2	37	−0.90	2.84 × 10^−9^
*Sus scrofa*	Cetartiodactyla		41	−1.47	0.20	−7.23	8.91 × 10^−9^	2	39	−0.90	7.84 × 10^−10^
*Lama pacos*	Cetartiodactyla		25	−1.16	0.27	−4.26	ns	2	23	−0.84	1.94 × 10^−5^
Node 1106	Perissodactyla	Perissodactyla	34	−1.30	0.17	−7.52	1.20 × 10^−8^	2	32	−0.88	6.94 × 10^−8^
Node 1108	Perissodactyla	Rhinocerotidae	24	−1.18	0.12	−10.20	5.55 × 10^−10^	0	24	−1.00	1.19 × 10^−7^
*Ceratotherium simum*	Perissodactyla		38	−1.65	0.16	−10.40	5.02 × 10^−12^	1	37	−0.95	2.84 × 10^−10^
*Rhinoceros unicornis*	Perissodactyla		23	−1.32	0.17	−7.77	9.47 × 10^−8^	0	23	−1.00	2.38 x 10^−7^
*Equus asinus*	Perissodactyla		19	−1.56	0.17	−8.98	4.56 × 10^−8^	0	19	−1.00	3.81 × 10^−6^
*Equus caballus*	Perissodactyla		40	−1.89	0.16	−11.50	3.29 × 10^−12^	0	40	−1.00	1.82 × 10^−12^
Node 1119	Carnivora	Carnivora	36	−1.24	0.20	−6.05	6.57 × 10^−7^	3	33	−0.83	2.27 × 10^−7^
Node 1211	Carnivora	*Ursus* (*sensu lato*)	24	−1.15	0.21	−5.51	1.32 × 10^−5^	1	23	−0.92	2.98 × 10^−6^
*Ursus maritimus*	Carnivora		23	−0.87	0.16	−5.55	1.42 × 10^−5^	3	20	−0.74	ns
*Canis lupus*	Carnivora		39	−1.60	0.42	−3.81	ns	3	36	−0.85	3.61 × 10^−8^
Node 1239	Carnivora	Felidae	19	−1.64	0.27	−5.98	1.17 × 10^−5^	0	19	−1.00	3.81 × 10^−6^
Node 1242	Carnivora	Along backbone of Felidae tree	19	−1.97	0.28	−7.11	1.25 × 10^−6^	0	19	−1.00	3.81 × 10^−6^
*Felis silvestris*	Carnivora		29	−1.46	0.19	−7.87	1.41 × 10^−8^	1	28	−0.93	1.12 × 10^−7^
*Acinonyx jubatus*	Carnivora		23	−1.37	0.18	−7.56	1.49 × 10^−7^	0	23	−1.00	2.38 × 10^−7^
Node 1332	Chiroptera	Chiroptera	40	−0.98	0.15	−6.53	9.43 × 10^−8^	6	34	−0.70	8.36 × 10^−6^
*Pteropus giganteus*	Chiroptera		16	−1.84	0.30	−6.09	2.06 × 10^−5^	2	14	−0.75	ns
*Megaderma lyra*	Chiroptera		19	−1.69	0.23	−7.28	9.14 × 10^−7^	0	19	−1.00	3.81 × 10^−6^
Node 1458	Chiroptera	*Rhinolophus*	23	−1.00	0.18	−5.51	1.54 × 10^−5^	2	21	−0.83	6.60 × 10^−5^
*Rhinolophus monoceros*	Chiroptera		18	−2.29	0.26	−8.88	8.60 × 10^−8^	0	18	−1.00	7.63 × 10^−6^
*Rhinolophus cornutus*	Chiroptera		15	−2.07	0.33	−6.30	1.95 × 10^−5^	1	14	−0.87	ns
*Tadarida brasiliensis*	Chiroptera		17	−2.06	0.25	−8.11	4.61 × 10^−7^	0	17	−1.00	1.53 × 10^−5^
*Talpa europaea*	Eulipotyphla		27	−1.19	0.17	−6.89	2.60 × 10^−7^	1	26	−0.93	4.17 × 10^−7^
*Sorex unguiculatus*	Eulipotyphla		23	−1.04	0.19	−5.51	1.53 × 10^−5^	1	22	−0.91	5.72 × 10^−6^
Node 1858	Xenarthra	Xenarthra	36	−1.02	0.18	−5.61	2.49 × 10^−6^	4	32	−0.78	1.94 × 10^−6^
*Tamandua tetradactyla*	Xenarthra		40	−1.02	0.16	−6.45	1.22 × 10^−7^	6	34	−0.70	8.36 × 10^−6^
*Myrmecophaga tridactyla*	Xenarthra		18	−1.72	0.27	−6.45	5.98 × 10^−6^	1	17	−0.89	ns
Node 1869	Xenarthra	*Chaetophractus* + *Euphractus* + *Zaedyus*	16	−1.74	0.38	−4.57	ns	0	16	−1.00	3.05 × 10^−5^
*Dasypus novemcinctus*	Xenarthra		28	−0.78	0.14	−5.47	8.60 × 10^−6^	4	24	−0.71	ns
Node 1878		Afrosoricida + Tubulidentata + Macroscelididae	21	2.71	0.27	9.87	3.95 × 10^−9^	21	0	1.00	9.54 × 10^−7^
*Chrysochloris asiatica*	Afrosoricida		25	−0.86	0.15	−5.75	6.32 × 10^−6^	2	23	−0.84	1.94 × 10^−5^
*Amblysomus hottentotus*	Afrosoricida		19	−1.78	0.24	−7.46	6.53 × 10^−7^	0	19	−1.00	3.81 × 10^−6^
*Orycteropus afer*	Tubulidentata		43	−1.50	0.18	−8.56	9.39 × 10^−11^	2	41	−0.91	2.15 × 10^−10^
Node 1908		Sirenia + Hyracoidea + Proboscidea	39	−1.04	0.17	−6.20	3.01 × 10^−7^	5	34	−0.74	2.43 × 10^−6^
*Dugong dugon*	Sirenia		28	−1.81	0.32	−5.64	5.43 × 10^−6^	0	28	−1.00	7.45 × 10^−9^
*Trichechus manatus*	Sirenia		20	−2.15	0.29	−7.53	4.07 × 10^−7^	0	20	−1.00	1.91 × 10^−6^
*Procavia capensis*	Hyracoidea		39	−0.89	0.16	−5.44	3.29 × 10^−6^	7	32	−0.64	7.03 × 10^−5^
*Loxodonta africana*	Proboscidea		43	−1.28	0.23	−5.53	1.90 × 10^−6^	12	31	−0.44	ns
Node 1924	Marsupialia	Diprotodontia less (Vombatidae + *Phascolarctos*)	29	−1.12	0.25	−4.56	ns	4	25	−0.72	1.04 × 10^−4^
*Trichosurus vulpecula*	Marsupialia		25	−1.19	0.18	−6.50	1.02 × 10^−6^	2	23	−0.84	1.94 × 10^−5^
*Dromiciops gliroides*	Marsupialia		19	−1.04	0.22	−4.64	ns	1	18	−0.90	7.63 × 10^−5^

**Table 3:** Branches identified as being significantly rate shifted compared to an ancestral branch no greater than three intervening branches removed. All *P*-values listed are significant at a nominal alpha of 0.05 corrected for multiple comparisons.

**Node**	**Order**	**Description**	**Reference ancestor**	**Depth to Paired ancestor*n***		**Average paired difference**	**SE**	**Paired*t***	**Paired*t P*-value**	n_+_	n_−_	**Proportion**	**Paired sign*P*-value**
Node 6		Boreoeutheria + Xenarthra	Node 5	1	16	3.16	0.36	8.84	2.48 × 10^−7^	16	0	1.00	3.05 × 10^−5^
Node 7		Boreoeutheria Myomorpha +	Node 6	1	19	−2.77	0.35	−7.92	2.83 × 10^−7^	0	19	−1.00	3.81 × 10^−6^
Node 11	Rodentia	Hystricomorpha Muridae +	Node 10	1	22	2.69	0.29	9.27	7.14 × 10^−9^	22	0	1.00	4.77 × 10^−7^
Node 19	Rodentia	Dipodidae Primates + Dermoptera +	Node 11	3	18	−2.69	0.29	−9.31	4.39 × 10^−8^	0	18	−1.00	7.63 × 10^−6^
Node 749		Scandentia	Node 8	1	19	2.26	0.27	8.39	1.24 × 10^−7^	18	1	0.90	7.63 × 10^−5^
*Cynocephalus variegatus*	Dermoptera	Cetartiodactyla + Perissodactyla + Carnivora +	Node 750	2	23	−0.93	0.25	−3.80		2	21	−0.83	6.60 × 10^−5^
Node 930		Pholidota Cetartiodactyla +	Node 928	2	19	2.36	0.37	6.38	5.27 × 10^−6^	16	3	0.68	ns
Node 931		Perissodactyla	Node 928	3	23	1.06	0.22	4.85	ns	21	2	0.83	6.60 × 10^−5^
Node 938	Cetartiodactyla	Bovidae Delphinidae +	Node 937	1	15	3.71	0.45	8.28	9.12 × 10^−7^	15	0	1.00	6.10 × 10^−5^
Node 1057	Cetartiodactyla	Phocoenidae	Node 1055	2	14	1.66	0.16	10.60	8.83 × 10^−8^	14	0	1.00	1.22 × 10^−4^
Node 1081	Cetartiodactyla	Physeteridae	Node 1051	2	21	−1.93	0.19	−10.10	2.89 × 10^−9^	1	20	−0.91	2.10 × 10^−5^
*Physeter catodon*	Cetartiodactyla	~ Balaenidae +	Node 1051	3	25	−2.09	0.15	−14.00	5.07 × 10^−13^	0	25	−1.00	5.96 × 10^−8^
Node 1083	Cetartiodactyla	Balaenopteridae	Node 1051	1	17	−2.18	0.17	−12.90	7.52 × 10^−10^	0	17	−1.00	1.53 × 10^−5^
*Caperea marginata*	Cetartiodactyla	*Baleonoptera* + *Megaptera* +	Node 1051	3	22	−2.06	0.12	−16.70	3.38 × 10^−12^	0	22	−1.00	4.77 × 10^−7^
Node 1087	Cetartiodactyla	*Eschrichtius*	Node 1051	2	16	−1.90	0.21	−9.12	1.67 × 10^−7^	0	16	−1.00	3.05 × 10^−5^
*Balaenoptera acutorostrata*	Cetartiodactyla		Node 1051	3	19	−2.26	0.19	−12.10	4.56 × 10^−10^	0	19	−1.00	3.81 × 10^−6^
Node 1106	Perissodactyla	Perissodactyla	Node 929	3	24	−1.31	0.22	−5.99	4.18 × 10^−6^	1	23	−0.92	2.98 × 10^−6^
Node 1409	Chiroptera	Microchiroptera Afrosoricida + Tubulidentata +	Node 1332	1	25	1.04	0.19	5.63	8.52 × 10^−6^	22	3	0.76	ns
Node 1878		Macroscelididae	Node 1877	1	19	3.08	0.35	8.77	6.41 × 10^−8^	19	0	1.00	3.81 × 10^−6^
*Orycteropus afer*	Tubulidentata		Node 1877	2	32	−1.17	0.16	−7.36	2.73 × 10^−8^	4	28	−0.75	1.93 × 10^−5^
Node 1923	Marsupialia	Diprotodontia	Node 1922	1	18	−2.22	0.35	−6.44	6.15 × 10^−6^	1	17	−0.89	1.45 × 10^−4^
*Dromiciops gliroides*	Marsupialia		Node 1922	1	18	−2.33	0.32	−7.33	1.17 × 10^−6^	0	18	−1.00	7.63 × 10^−6^

**Table 4:** Branches identified as being significantly rate shifted compared to their immediately ancestral branch (i.e. depth to ancestor = 1). All *P*-values listed are significant at a nominal alpha of 0.05 corrected for multiple comparisons.

**Node**	**Order**	**Description**	**Reference ancestor**	**Paired*n***	**Average paired difference**	**SE**	**Paired*t***	**Paired*t P*-value**	*n*_+_	*n*_−_	**Proportion**	**Paired sign*P*-value**
Node 6		Boreoeutheria + Xenarthra	Node 5	16	3.16	0.36	8.84	2.48 × 10^−7^	16	0	1.00	3.05 × 10^−5^
Node 7		Boreoeutheria	Node 6	19	−2.77	0.35	−7.92	2.83 × 10^−7^	0	19	−1.00	3.81 × 10^−6^
Node 11	Rodentia	Myomorpha + Hystricomorpha	Node 10	22	2.69	0.29	9.27	7.14 × 10^−9^	22	0	1.00	4.77 × 10^−7^
Node 12	Rodentia	Myomorpha	Node 11	15	−2.10	0.26	−8.05	1.28 × 10^−6^	1	14	−0.87	ns
Node 749		Primates + Dermoptera + Scandentia	Node 8	19	2.26	0.27	8.39	1.24 × 10^−7^	18	1	0.90	7.63 × 10^−5^
Node 750		Primates + Dermoptera	Node 749	15	−2.22	0.22	−10.30	6.85 × 10^−8^	0	15	−1.00	6.10 × 10^−5^
Node 930		Cetartiodactyla + Perissodactyla + Carnivora + Pholidota	Node 929	18	2.10	0.26	8.07	3.24 × 10^−7^	17	1	0.89	1.45 × 10^−4^
Node 932	Cetartiodactyla	Cetartiodactyla	Node 931	22	−1.04	0.19	−5.42	2.22 × 10^−5^	2	20	−0.82	1.21 × 10^−4^
Node 938	Cetartiodactyla	Bovidae	Node 937	15	3.71	0.45	8.28	9.12 × 10^−7^	15	0	1.00	6.10 × 10^−5^
Node 1083	Cetartiodactyla	~ Balaenidae + Balaenopteridae	Node 1051	17	−2.18	0.17	−12.90	7.52 × 10^−10^	0	17	−1.00	1.53 × 10^−5^
Node 1106	Perissodactyla	Perissodactyla	Node 931	21	−1.48	0.17	−8.67	3.32 × 10^−8^	0	21	−1.00	9.54 × 10^−7^
*Equus caballus*	Perissodactyla		Node 1114	23	−1.18	0.20	−5.95	5.46 × 10^−6^	2	21	−0.83	6.60 × 10^−5^
Node 1332	Chiroptera	Chiroptera	Node 929	25	−0.93	0.18	−5.30	1.97 × 10^−5^	5	20	−0.60	ns
Node 1409	Chiroptera	Microchiroptera	Node 1332	25	1.04	0.19	5.63	8.52 × 10^−6^	22	3	0.76	1.57 × 10^−4^
Node 1858	Xenarthra	Xenarthra	Node 6	19	−3.65	0.22	−16.80	1.91 × 10^−12^	0	19	−1.00	3.81 × 10^−6^
Node 1878		Afrosoricida + Tubulidentata + Macroscelididae	Node 1877	19	3.08	0.35	8.77	6.41 × 10^−8^	19	0	1.00	3.81 × 10^−6^
Node 1879		Afrosoricida + Macroscelididae	Node 1878	13	−2.49	0.37	−6.66	2.34 × 10^−5^	0	13	−1.00	ns
*Orycteropus afer*	Tubulidentata		Node 1878	21	−3.92	0.22	−18.20	6.68 × 10^−14^	0	21	−1.00	9.54 × 10^−7^
Node 1923	Marsupialia	Diprotodontia	Node 1922	18	−2.22	0.35	−6.44	6.15 × 10^−6^	1	17	−0.89	1.45 × 10^−4^
*Dromiciops gliroides*	Marsupialia		Node 1922	18	−2.33	0.32	−7.33	1.17 × 10^−6^	0	18	−1.00	7.63 × 10^−6^

**Table 5:** Clades identified as being significant rate outliers compared to mammals as a whole. All *P*-values listed are significant at a nominal alpha of 0.05 corrected for multiple comparisons.

**Node**	**Order**	**Description**	**Paired*n***	**Average-paired difference**	**SE**	**Paired*t***	**Paired*t P*-value**	*n*_+_	n_−_	**Proportion**	**Paired sign*P*-value**
Node 2	Monotremata		17	−1.11	0.14	−7.79	7.86 × 10^−7^	0	17	−1.00	1.53 × 10^−5^
Node 3	Monotremata	Tachyglossidae	15	−1.09	0.20	−5.51	7.71 × 10^−5^	0	15	−1.00	6.10 × 10^−5^
Node 4		Theria	42	0.31	0.07	4.34	ns	17	0	1.00	1.53 × 10^−5^
Node 5		Eutheria	44	0.92	0.12	7.66	1.46 × 10^−9^	38	3	0.85	1.05 × 10^−8^
Node 464	Rodentia	Sciuromorpha	42	−0.81	0.15	−5.23	5.29 × 10^−6^	7	35	−0.67	1.51 × 10^−5^
Node 673	Lagomorpha	Lagomorpha Primates +	43	−0.83	0.18	−4.66	3.21 × 10^−5^	9	34	−0.58	1.70 × 10^−4^
Node 750		Dermoptera	44	−0.65	0.14	−4.70	2.68 × 10^−5^	12	32	−0.46	ns
Node 816	Primates	*Homo* + *Pan*	35	−1.27	0.19	−6.53	1.78 × 10^−7^	4	31	−0.77	3.47 × 10^−6^
Node 817	Primates	*Pan*	23	−0.99	0.15	−6.43	1.79 × 10^−6^	3	20	−0.74	ns
Node 888	Primates	Strepsirrhini Cetartiodactyla +	41	−1.16	0.15	−7.69	2.11 × 10^−9^	3	38	−0.85	1.05 × 10^−8^
Node 931		Perissodactyla	44	−0.93	0.13	−7.26	5.45 × 10^−9^	5	39	−0.77	1.41 × 10^−7^
Node 938	Cetartiodactyla	Bovidae *Ovis* + *Hemitragus*	26	−0.64	0.15	−4.30	ns	3	23	−0.77	8.80 × 10^−5^
Node 954	Cetartiodactyla	+ *Capra* + *Pseudois Bos* + *Bison* + *Bubalus*	21	−1.07	0.20	−5.32	3.28 × 10^−5^	2	19	−0.81	2.21 × 10^−4^
Node 1006	Cetartiodactyla	*Syncerus* Cervinae *+*	26	−0.78	0.12	−6.34	1.24 × 10^−6^	1	25	−0.92	8.05 × 10^−7^
Node 1035	Cetartiodactyla	Muntacinae	19	−0.86	0.13	−6.83	2.14 × 10^−6^	0	19	−1.00	3.81 × 10^−6^
Node 1042	Cetartiodactyla	*Muntiacus* Delphinidae +	26	−0.85	0.13	−6.35	1.19 × 10^−6^	2	24	−0.85	1.05 × 10^−5^
Node 1057	Cetartiodactyla	Phocoenidae	25	−0.93	0.26	−3.59	ns	2	23	−0.84	1.94 × 10^−5^
Node 1079	Cetartiodactyla	Ziphiidae	27	−1.42	0.25	−5.72	5.11 × 10^−6^	2	25	−0.85	5.65 × 10^−6^
Node 1081	Cetartiodactyla	Physeteridae ~ Balaenidae +	25	−1.29	0.19	−6.75	5.60 × 10^−7^	0	25	−1.00	5.96 × 10^−8^
Node 1083	Cetartiodactyla	Balaenopteridae Balaenidae +	26	−0.96	0.17	−5.51	1.01 × 10^−5^	2	24	−0.85	1.05 × 10^−5^
Node 1084	Cetartiodactyla	*Caperea Baleonoptera* + *Megaptera* +	24	−1.36	0.17	−8.20	2.79 × 10^−8^	1	23	−0.92	2.98 × 10^−6^
Node 1087	Cetartiodactyla	*Eschrichtius Baleonoptera* (less *B. acutorostrata*) +	24	−1.06	0.21	−5.07	3.96 × 10^−5^	3	21	−0.75	ns
Node 1088	Cetartiodactyla	*Megaptera* +*Eschrichtius Baleonoptera physalus* + *Baleonoptera musculus* + *Megaptera*	26	−1.27	0.26	−4.96	4.09 × 10^−5^	4	22	−0.69	ns
Node 1090	Cetartiodactyla	*Baleonoptera*	23	−1.48	0.15	−10.10	1.09 × 10^−9^	1	22	−0.91	5.72 × 10^−6^
Node 1106	Perissodactyla	Perissodactyla Rhinocerotidae +	41	−1.43	0.15	−9.34	1.34 × 10^−11^	1	40	−0.95	3.82 × 10^−11^
Node 1107	Perissodactyla	Tapiridae	18	−1.78	0.28	−6.36	7.14 × 10^−6^	1	17	−0.89	1.45 × 10^−4^
Node 1108	Perissodactyla	Rhinocerotidae	25	−1.25	0.14	−8.78	5.80 × 10^−9^	0	25	−1.00	5.96 × 10^−8^
Node 1114	Perissodactyla	Equidae Carnivora +	24	−1.66	0.16	−10.20	5.55 × 10^−10^	0	24	−1.00	1.19 × 10^−7^
Node 1118		Pholidota	28	−0.74	0.14	−5.34	1.23 × 10^−5^	3	25	−0.79	2.74 × 10^−5^
Node 1119	Carnivora	Carnivora	42	−1.08	0.14	−7.65	2.06 × 10^−9^	4	38	−0.81	5.65 × 10^−8^
Node 1120	Carnivora	Caniformia	39	−1.01	0.15	−6.93	3.08 × 10^−8^	3	36	−0.85	3.61 × 10^−8^
Node 1121	Carnivora	Arctoidea	29	−0.76	0.14	−5.36	1.04 × 10^−5^	6	23	−0.59	ns
Node 1211	Carnivora	*Ursus* (*sensu lato*)	26	−1.07	0.21	−5.00	3.73 × 10^−5^	2	24	−0.85	1.05 × 10^−5^
Node 1239	Carnivora	Felidae	21	−1.21	0.14	−8.71	3.07 × 10^−8^	0	21	−1.00	9.54 × 10^−7^
Node 1243	Carnivora	Along backbone of Felidae	17	−1.89	0.30	−6.22	1.23 × 10^−5^	0	17	−1.00	1.53 × 10^−5^
Node 1332	Chiroptera	Chiroptera	44	−0.74	0.16	−4.61	3.55 × 10^−5^	9	35	−0.59	1.06 × 10^−4^
Node 1409	Chiroptera	Microchiroptera	43	−0.92	0.15	−6.27	1.62 × 10^−7^	8	35	−0.63	4.19 × 10^−5^
Node 1458	Chiroptera	*Rhinolophus*	25	−1.29	0.27	−4.82	ns	2	23	−0.84	1.94 × 10^−5^
Node 1494	Chiroptera	Major clade in Microchiroptera	41	−0.85	0.16	−5.33	4.16 × 10^−6^	7	34	−0.66	2.53 × 10^−5^
Node 1762	Eulipotyphla	Eulipotyphla less Soleonodontidae	43	−0.46	0.14	−3.35	ns	9	34	−0.58	1.70 × 10^−4^
Node 1766	Eulipotyphla	Talpinae	38	−1.04	0.17	−6.04	5.48 × 10^−7^	6	32	−0.68	2.43 × 10^−5^
Node 1770	Eulipotyphla	*Mogera* + *Euroscaptor* +*Talpa*	27	−0.98	0.19	−5.28	1.63 × 10^−5^	2	25	−0.85	5.65 × 10^−6^
Node 1858	Xenarthra	Xenarthra	41	−1.04	0.15	−6.94	2.29 × 10^−8^	4	37	−0.81	1.03 × 10^−7^
Node 1864	Xenarthra	*Tamandua* + *Myrmecophaga*	18	−1.49	0.27	−5.55	3.56 × 10^−5^	2	16	−0.78	ns
Node 1880	Afrosoricida	Afrosoricida	41	−0.78	0.12	−6.55	8.10 × 10^−8^	5	36	−0.76	7.84 × 10^−7^
Node 1909		Sirenia + Hyracoidea	40	−1.16	0.16	−7.36	6.97 × 10^−9^	4	36	−0.80	1.86 × 10^−7^
Node 1918	Marsupialia	Marsupialia	33	−1.06	0.17	−6.19	6.22 × 10^−7^	2	31	−0.88	1.31 × 10^−7^
Node 1924	Marsupialia	Diprotodontia less (Vombatidae + *Phascolarctos*)	30	−0.97	0.18	−5.49	6.46 × 10^−6^	3	27	−0.80	8.43 × 10^−6^

**Table 6:** Clades identified as being significantly rate shifted compared to an ancestral clade no greater than three intervening branches removed. All *P*-values listed are significant at a nominal alpha of 0.05 corrected for multiple comparisons.

**Node**	**Order**	**Description**	**Paired*n***	**Reference ancestor**	**Depth to ancestor**	**Average paired difference**	**SE**	**Paired*t***	**Paired*t P*-value**	***n*_+_**	***n*_–_**	**Proportion**	**Paired sign*P*-value**
Node 2	Monotremata		17	Node 1	1	−1.11	0.14	−7.79	7.86 × 10^−7^	0	17	−1.00	1.53 × 10^−5^
Node 4		Theria	17	Node 1	1	0.78	0.10	7.51	1.26 × 10^−6^	17	0	1.00	1.53 × 10^−5^
Node 5		Eutheria Boreoeutheria +	42	Node 4	1	0.65	0.07	8.89	4.48 × 10^−11^	38	3	0.85	1.05 × 10^−8^
Node 6		Xenarthra	44	Node 5	1	−1.02	0.15	−6.89	1.83 × 10^−8^	10	34	−0.55	ns
Node 7		Boreoeutheria	44	Node 6	1	0.34	0.07	4.69	2.77 × 10^−5^	34	10	0.55	ns
Node 10	Rodentia	Rodentia Myomorpha +	44	Node 9	1	0.44	0.08	5.35	3.19 × 10^−6^	35	9	0.59	1.06 × 10^−4^
Node 11	Rodentia	Hystricomorpha	44	Node 10	1	−0.64	0.14	−4.62	3.45 × 10^−5^	13	31	−0.41	ns
Node 464	Rodentia	Sciuromorpha	42	Node 10	1	−1.25	0.15	−8.22	3.33 × 10^−10^	5	37	−0.76	4.43 × 10^−7^
Node 673	Lagomorpha	Lagomorpha Primates + Dermoptera +	43	Node 9	1	−0.77	0.13	−5.72	1.02 × 10^−6^	4	39	−0.81	3.11 × 10^−8^
Node 749		Scandentia	40	Node 8	1	−1.08	0.12	−9.09	3.85 × 10^−11^	0	40	−1.00	1.82 × 10^−12^
Node 750		Primates +Dermoptera	44	Node 8	2	−1.09	0.13	−8.12	3.29 × 10^−10^	5	39	−0.77	1.41 × 10^−7^
Node 815	Primates	*Homo* + *Pan* +Gorilla	27	Node 754	3	−0.85	0.13	−6.42	8.44 × 10^−7^	3	24	−0.78	4.92 × 10^−5^
Node 888	Primates	Strepsirrhini Cetartiodactyla +	41	Node 751	1	−0.48	0.09	−5.10	8.49 × 10^−6^	8	33	−0.61	1.12 × 10^−4^
Node 931		Perissodactyla	44	Node 930	1	−0.45	0.08	−5.66	1.15 × 10^−6^	11	33	−0.50	ns
Node 932	Cetartiodactyla	Cetartiodactyla	42	Node 931	1	0.39	0.08	4.87	1.73 × 10^−5^	34	8	0.62	6.88 × 10^−5^
Node 938	Cetartiodactyla	Bovidae *Bos* + *Bison* + *Bubalus* +	25	Node 937	1	−1.78	0.27	−6.57	8.48 × 10^−7^	3	22	−0.76	1.57 × 10^−4^
Node 1006	Cetartiodactyla	*Syncerus* Cervinae +	26	Node 937	3	−1.85	0.30	−6.25	1.56 × 10^−6^	4	22	−0.69	ns
Node 1035	Cetartiodactyla	Muntacinae Delphinidae + Phocoenidae + Monodontidae +	19	Node 937	3	−2.23	0.30	−7.44	6.81 × 10^−7^	0	19	−1.00	3.81 × 10^−6^
Node 1055	Cetartiodactyla	Platanistidae (in part)	28	Node 1054	1	0.28	0.09	3.01	ns	24	4	0.71	1.80 × 10^−4^
Node 1079	Cetartiodactyla	Ziphiidae	27	Node 1054	1	−0.72	0.16	−4.40	1.65 × 10^−4^	3	24	−0.78	4.92 × 10^−5^
Node 1081	Cetartiodactyla	Physeteridae ~ Balaenidae +	25	Node 1052	1	−1.11	0.22	−5.06	3.53 × 10^−5^	3	22	−0.76	1.57 × 10^−4^
Node 1083	Cetartiodactyla	Balaenopteridae *Baleonoptera* (less *B. acutorostrata*)+ *Megaptera* +	26	Node 1051	1	−1.21	0.21	−5.72	5.90 × 10^−6^	3	23	−0.77	8.80 × 10^−5^
Node 1088	Cetartiodactyla	*Eschrichtius* *Baleonoptera physalus* +*Baleonoptera musculus* +	26	Node 1051	3	−1.48	0.27	−5.53	9.60 × 10^−6^	4	22	−0.69	ns
Node 1090	Cetartiodactyla	*Megaptera*	23	Node 1088	1	−0.61	0.13	−4.74	ns	2	20	−0.82	1.21 × 10^−4^
Node 1106	Perissodactyla	Perissodactyla	41	Node 931	1	−0.56	0.08	−7.01	1.83 × 10^−8^	3	38	−0.85	1.05 × 10^−8^
Node 1114	Perissodactyla	Equidae	24	Node 1106	1	−0.64	0.10	−6.18	2.64 × 10^−6^	1	23	−0.92	2.98 × 10^−6^
Node 1119	Carnivora	Carnivora	42	Node 930	2	−0.63	0.10	−6.47	9.31 × 10^−8^	6	36	−0.71	2.83 × 10^−6^
Node 1120	Carnivora	Caniformia *Eumetopias* +*Otaria* + *Neophoca* +	39	Node 930	3	−0.67	0.11	−5.89	8.17 × 10^−7^	5	34	−0.74	2.43 × 10^−6^
Node 1190	Carnivora	*Phocarctos*	11	Node 1184	2	0.83	0.10	8.06	1.10 × 10^−5^	11	0	1.00	ns
Node 1332	Chiroptera	Chiroptera Eulipotyphla less	44	Node 929	1	−0.83	0.14	−5.81	6.93 × 10^−7^	8	36	−0.64	2.54 × 10^−5^
Node 1762	Eulipotyphla	Soleonodontidae	43	Node 928	2	−0.59	0.12	−5.01	1.03 × 10^−5^	9	34	−0.58	1.70 × 10^−4^
Node 1766	Eulipotyphla Erinaceidae +	Talpinae	38	Node 1762	3	−0.71	0.11	−6.41	1.75 × 10^−7^	8	30	−0.58	ns
Node 1785	Eulipotyphla	Soricidae	42	Node 928	3	−0.59	0.11	−5.32	4.01 × 10^−6^	7	35	−0.67	1.51 × 10^−5^
Node 1858	Xenarthra	Xenarthra*Tamandua* +	41	Node 6	1	−0.99	0.09	−11.20	7.02 × 10^−14^	2	39	−0.90	7.84 × 10^−10^
Node 1864	Xenarthra	Myrmecophaga Afrosoricida + Tubulidentata +	18	Node 6	3	−1.03	0.13	−8.09	3.14 × 10^−7^	0	18	−1.00	7.63 × 10^−6^
Node 1878		Macroscelididae	43	Node 1877	1	−0.89	0.19	−4.71	2.75 × 10^−5^	13	30	−0.40	ns
Node 1880	Afrosoricida	Afrosoricida Sirenia + Hyracoidea +	41	Node 1877	3	−1.16	0.20	−5.83	8.30 × 10^−7^	9	32	−0.56	ns
Node 1908		Proboscidea	44	Node 1877	1	−1.05	0.15	−6.99	1.32 × 10^−8^	6	38	−0.73	9.43 × 10^−7^
Node 1917	Proboscidea	Proboscidea Diprotodontia + Vombatidae + *Dromiciops* + Dasyuromorpha + Notoryctemorphia +	9	Node 1908	1	−1.64	0.15	−10.90	4.44 × 10^−6^	0	9	−1.00	ns
Node 1920	Marsupialia	Peramelemorphia	29	Node 4	3	−0.94	0.15	−6.32	7.85 × 10−7	1	28	−0.93	1.12 × 10^−7^

**Table 7:** Clades identified as being significantly rate shifted compared to their immediately ancestral clade (i.e. depth to ancestor = 1). All *P*-values listed are significant at a nominal alpha of 0.05 corrected for multiple comparisons.

**Node**	**Order**	**Description**	**Reference ancestor**	**Depth to ancestor**	**Paired*n***	**Average paired difference**	**SE**	**Paired*t***	**Paired*t P*-value**	n_+_	n_−_	**Proportion**	**Paired sign*P*-value**
Node 2	Monotremata		Node 1	1	17	−1.11	0.14	−7.79	7.86 × 10^−7^	0	17	−1.00	1.53 × 10^−5^
Node 4		Theria	Node 1	1	17	0.78	0.10	7.51	1.26 × 10^−6^	17	0	1.00	1.53 × 10^−5^
Node 5		Eutheria	Node 4	1	42	0.65	0.07	8.89	4.48 × 10^−11^	38	3	0.85	1.05 × 10^−8^
		Boreoeutheria +											
Node 6		Xenarthra	Node 5	1	44	−1.02	0.15	−6.89	1.83 × 10^−8^	10	34	−0.55	ns
Node 7		Boreoeutheria	Node 6	1	44	0.34	0.07	4.69	2.77 × 10^−5^	34	10	0.55	ns
Node 10	Rodentia	Rodentia	Node 9	1	44	0.44	0.08	5.35	3.19 × 10^−6^	35	9	0.59	1.06 × 10^−4^
		Myomorpha +											
Node 11	Rodentia	Hystricomorpha	Node 10	1	44	−0.64	0.14	−4.62	3.45 × 10^−5^	13	31	−0.41	ns
Node 464	Rodentia	Sciuromorpha	Node 10	1	42	−1.25	0.15	−8.22	3.33 × 10^−10^	5	37	−0.76	4.43 × 10^−7^
Node 673	Lagomorpha	Lagomorpha	Node 9	1	43	−0.77	0.13	−5.72	1.02 × 10^−6^	4	39	−0.81	3.11 × 10^−8^
		Primates +											
		Dermoptera +											
Node 749		Scandentia	Node 8	1	40	−1.08	0.12	−9.09	3.85 × 10^−11^	0	40	−1.00	1.82 × 10^−12^
		*Homo* + *Pan* +											
Node 815	Primates	*Gorilla*	Node 814	1	25	−0.53	0.10	−5.12	3.06 × 10^−5^	4	21	−0.68	ns
Node 888	Primates	Strepsirrhini	Node 751	1	41	−0.48	0.09	−5.10	8.49 × 10^−6^	8	33	−0.61	1.12 × 10^−4^
		Cetartiodactyla +											
Node 931		Perissodactyla	Node 930	1	44	−0.45	0.08	−5.66	1.15 × 10^−6^	11	33	−0.50	ns
Node 932	Cetartiodactyla	Cetartiodactyla	Node 931	1	42	0.39	0.08	4.87	1.73 × 10^−5^	34	8	0.62	6.88 × 10^−5^
Node 938	Cetartiodactyla	Bovidae	Node 937	1	25	−1.78	0.27	−6.57	8.48 × 10^−7^	3	22	−0.76	1.57 × 10^−4^
		Delphinidae +											
		Phocoenidae +											
		Monodontidae +											
Node 1055	Cetartiodactyla	Platanistidae (in part)	Node 1054	1	28	0.28	0.09	3.01	ns	24	4	0.71	1.80 × 10^−4^
Node 1079	Cetartiodactyla	Ziphiidae	Node 1054	1	27	−0.72	0.16	−4.40	ns	3	24	−0.78	4.92 × 10^−5^
Node 1081	Cetartiodactyla	Physeteridae	Node 1052	1	25	−1.11	0.22	−5.06	3.53 × 10^−5^	3	22	−0.76	1.57 × 10^−4^
		~ Balaenidae +											
Node 1083	Cetartiodactyla	Balaenopteridae	Node 1051	1	26	−1.21	0.21	−5.72	5.90 × 10^−6^	3	23	−0.77	8.80 × 10^−5^
		*Baleonoptera physalus* +											
		*Baleonoptera musculus* +											
Node 1090	Cetartiodactyla	*Megaptera*	Node 1088	1	23	−0.61	0.13	−4.74	ns	2	20	−0.82	1.21 × 10^−4^
Node 1114	Perissodactyla	Equidae	Node 1106	1	24	−0.64	0.10	−6.18	2.64 × 10^−6^	1	23	−0.92	2.98 × 10^−6^
Node 1332	Chiroptera	Chiroptera	Node 929	1	44	−0.83	0.14	−5.81	6.93 × 10^−7^	8	36	−0.64	2.54 × 10^−5^
Node 1786	Eulipotyphla	Erinaceidae	Node 1785	1	27	0.26	0.07	3.82	ns	23	4	0.70	3.11 × 10^−4^
Node 1858	Xenarthra	Xenarthra	Node 6	1	41	−0.99	0.09	−11.20	7.02 × 10^−14^	2	39	−0.90	7.84 × 10^−10^
		Afrosoricida +											
		Tubulidentata +											
Node 1878		Macroscelididae	Node 1877	1	43	−0.89	0.19	−4.71	2.75 × 10^−5^	13	30	−0.40	ns
		Sirenia + Hyracoidea +											
Node 1908		Proboscidea	Node 1877	1	44	−1.05	0.15	−6.99	1.32 × 10^−8^	6	38	−0.73	9.43 × 10^−7^
Node 1917	Proboscidea	Proboscidea	Node 1908	1	9	−1.64	0.15	−10.90	4.44 × 10^−6^	0	9	−1.00	ns
Node 1918	Marsupialia	Marsupialia	Node 4	1	33	−1.46	0.14	−10.50	6.82 × 10^−12^	0	33	−1.00	2.33 × 10^−10^
